# Supplementary material for: Estimating the real burden of disease under a pandemic situation: The SARS-CoV2 case
Source: PLoS One. 2020 Dec 3;15(12):e0242956. doi: 10.1371/journal.pone.0242956 (PMC7714127; doi:10.1371/journal.pone.0242956)
Supplement: S1 Appendix — (PDF) [file pone.0242956.s001.pdf]

S1 Appendix

The SIR model described is defined by the system of differential equations:

$$\frac{\mathrm{d}S(t)}{\mathrm{d}t} = -\beta \frac{I(t)S(t)}{N} \tag{S1.1}$$

$$\frac{\mathrm{d}I(t)}{\mathrm{d}t} = \beta \frac{I(t)S(t)}{N} - \gamma I(t) \tag{S1.2}$$

$$\frac{\mathrm{d}R(t)}{\mathrm{d}t} = \gamma I(t) \tag{S1.3}$$

where $S(t)$ is the number of Susceptible individuals, $I(t)$ the number of Infectious and $R(t)$ the number of Removed (either recovered or deceased) individuals at time t. For each t, $S(t) + I(t) + R(t) = N$, where N is the total susceptible population, and it is a parameter to be estimated jointly with β and γ, that is, the infection and recovery rates.

Observe that by adding equations (S1.2) and (S1.3), and using the identities $S(t) = N - A(t)$ and $I(t) = A(t) - R(t)$, a simpler system of equations is obtained:

$$\frac{\mathrm{d}A(t)}{\mathrm{d}t} = \frac{\beta}{N} \left(N - A(t)\right) \left(A(t) - R(t)\right) \tag{S1.4}$$

$$\frac{\mathrm{d}R(t)}{\mathrm{d}t} = \gamma(A(t) - R(t))$$

where A_0 and R_0 are the number of affected and recovered individuals at time $n = 0$, respectively, so that

$$\frac{\mathrm{d}R}{\mathrm{d}A} = \frac{\mathrm{d}R}{\mathrm{d}t}\frac{\mathrm{d}t}{\mathrm{d}A} = \frac{\gamma}{\beta}\frac{N}{N - A(t)}. \tag{S1.5}$$

This is a separable variables equation, with solution

$$R(t) = \frac{N\gamma}{\beta} \log\left(\frac{N - A_0}{N - A(t)}\right) + R_0 \tag{S1.6}$$

that can be plugged in (S1.4) to get

$$\frac{\mathrm{d}A(t)}{\mathrm{d}t} = \frac{\beta}{N}\left(A(t) - \frac{N\gamma}{\beta}\log\left(\frac{N - A_0}{N - A(t)}\right) - R_0\right)(N - A(t)) \tag{S1.7}$$

A second-order Taylor approximation of the right-hand side of equation (S1.7) gives: $\frac{dA(t)}{\partial t} = kA\left(1 - \frac{A}{M^*}\right)$, where $k = \beta - \gamma$ and $M^* = \frac{N(\beta-\gamma)}{\beta-\gamma/2}$. Recall that β is the infection rate, γ is the recovery rate and N the total susceptible population.

The solution of this last equation is the logistic function:

$$A(t) = \frac{M^* A_0 \mathrm{e}^{kt}}{M^* + A_0(\mathrm{e}^{kt} - 1)}. \tag{S1.8}$$

In our analysis, the values of A_0 and R_0 have been fixed to one and zero respectively.

The maximum value of $A(t)$, that is $A(\infty) = A_\infty$, can be obtained equating to zero the left part of (S1.7). The maximum could be obtained solving numerically the equation,

$$A_\infty - \frac{N\gamma}{\beta} \log(\frac{N - A_0}{N - A_\infty}) + R_0 = 0. \tag{S1.9}$$

To estimate the three parameters of the SIR model, β, γ and N, we have used the equation $\hat{k} = \hat{\beta} - \hat{\gamma}$, the estimation $A_\infty \approx \hat{M}^*/(1 - \hat{\alpha})$ (see S2 Appendix) and the equation (S1.9). We need a third equation that can be the number of affected people A^* when $A(t)$ grows faster, undoubtedly an important moment of the outbreak. It can be found calculating the second derivative of $A(t)$ and equating to zero, obtaining ($R_0 = 0$),

$$-2\beta A^* + N(\gamma \log(\frac{N - A_0}{N - A^*} + \beta - \gamma) = 0. \tag{S1.10}$$

Combining the three equations we reduce them to only one, which can be used for estimating N:

$$A_\infty(\log(\frac{N - A_0}{N - A^*}) - 1) = (2A^* - N) \log(\frac{N - A_0}{N - A_\infty})\,. \tag{S1.11}$$

Then, β and γ can be obtained from the other equations.
