# Supplementary material for: Estimating the real burden of disease under a pandemic situation: The SARS-CoV2 case
Source: PLoS One. 2020 Dec 3;15(12):e0242956. doi: 10.1371/journal.pone.0242956 (PMC7714127; doi:10.1371/journal.pone.0242956)
Supplement: S2 Appendix — (PDF) [file pone.0242956.s002.pdf]

S2 Appendix

The latent process $\{X_n : n \geq 0\}$ is assumed to be INAR(1) given by $X_{n+1} = \alpha \circ X_n + W_n$, where $W_n \sim \text{Poisson}(\lambda_n)$, independent of X_n. The expectation of X_{n+1} is computed as $\mathrm{E}(X_{n+1}) = \alpha \mathrm{E}(X_n) + \lambda_{n+1}$ Therefore, the following representation can be derived:

$$\begin{aligned}
\mathrm{E}(X_{n+2}) &= \alpha\mathrm{E}(X_{n+1}) + \lambda_{n+2} = \alpha\left(\alpha\mathrm{E}(X_n) + \lambda_{n+1}\right) + \lambda_{n+2} = \alpha^2\mathrm{E}(X_n) + \alpha\lambda_{n+1} + \lambda_{n+2} \\
\mathrm{E}(X_{n+3}) &= \alpha\mathrm{E}(X_{n+2}) + \lambda_{n+3} = \alpha(\alpha^2\mathrm{E}(X_n) + \alpha\lambda_{n+1} + \lambda_{n+2}) + \lambda_{n+3} \\
&= \alpha^3\mathrm{E}(X_n) + \alpha^2\lambda_{n+1} + \alpha\lambda_{n+2} + \lambda_{n+3} \\
&\vdots \\
\mathrm{E}(X_{n+k}) &= \alpha^k\mathrm{E}(X_n) + \alpha^{k-1}\lambda_{n+1} + \alpha^{k-2}\lambda_{n+2} + \cdots + \lambda_{n+k} = \alpha^k\mathrm{E}(X_n) + \sum_{i=1}^{k}\alpha^{k-i}\lambda_{n+i}
\end{aligned} \tag{S2.1}$$

On the other hand, $\mathrm{E}(X_n) \approx \frac{Y_n}{1-\omega(1-q_n)}$ since we can assume that $\mathrm{E}(Y_n) \approx Y_n$ and we know that $\mathrm{E}(Y_{n+k}) = \mathrm{E}(X_{n+k})(1 - \omega(1 - q_{n+k}))$. Hence:

$$\begin{aligned}
\mathrm{E}(Y_{n+1}) &= \frac{1-\omega(1-q_{n+1})}{1-\omega(1-q_n)}\alpha Y_n + \left(1-\omega(1-q_{n+1})\right)\lambda_{n+1} \\
\mathrm{E}(Y_{n+2}) &= \frac{1-\omega(1-q_{n+2})}{1-\omega(1-q_n)}\alpha^2 Y_n + \left(1-\omega(1-q_{n+2})\right)\left(\alpha\lambda_{n+1} + \lambda_{n+2}\right) \\
&\vdots \\
\mathrm{E}(Y_{n+k}) &= \frac{1-\omega(1-q_{n+k})}{1-\omega(1-q_n)}\alpha^k Y_n + \left(1-\omega(1-q_{n+k})\right)\sum_{i=1}^{k}\alpha^{k-i}\lambda_{n+i}.
\end{aligned} \tag{S2.2}$$
