# Supplementary material for: Estimating the real burden of disease under a pandemic situation: The SARS-CoV2 case
Source: PLoS One. 2020 Dec 3;15(12):e0242956. doi: 10.1371/journal.pone.0242956 (PMC7714127; doi:10.1371/journal.pone.0242956)
Supplement: S3 Appendix — (PDF) [file pone.0242956.s003.pdf]

S3 Appendix

Assume $1_n \sim \text{Bernoulli}(\omega)$. The process Y_{n+k} is equal to X_{n+k} when $1_{n+k} = 0$, and $Y_{n+k} = q_{n+k} \circ X_{n+k}$ when $1_{n+k} = 1$. Therefore, the distribution of Y_{n+k} can be computed in two separate parts.

If $1_{n+k} = 1$, that is, if the observation Y_{n+k} is under-reported, its distribution takes the following form:

$$\begin{aligned}
Y_{n+1} &= q_{n+1} \circ X_{n+1} = q_{n+1} \left(\alpha \circ X_n + W(\lambda_{n+1})\right) \\
&= (\alpha q_{n+1}) \circ X_n + W(q_{n+1}\lambda_{n+1}) \\
Y_{n+2} &= q_{n+2} \circ \left(\alpha \circ (\alpha \circ X_n + W(\lambda_{n+1})) + W(\lambda_{n+2})\right) \\
&= \left(q_{n+2}\alpha^2\right) \circ X_n + W(\alpha q_{n+2}\lambda_{n+1}) + W(q_{n+2}\lambda_{n+2}) \\
Y_{n+3} &= q_{n+3} \circ X_{n+3} \\
&= q_{n+3} \left(\alpha \circ (\alpha \circ (\alpha \circ X_n + W(\lambda_{n+1})) + W(\lambda_{n+2})) + W(\lambda_{n+3})\right) \\
&= \left(q_{n+3}\alpha^3\right) \circ X_n + W(q_{n+3}\alpha^2\lambda_{n+1}) + W(q_{n+3}\alpha\lambda_{n+2}) + W(q_{n+3}\lambda_{n+3}) \\
&\vdots \\
Y_{n+k} &= (q_{n+k}\alpha^k) \circ X_n + W\left(q_{n+k}\sum_{i=1}^{k}\alpha^{k-i}\lambda_{n+i}\right). \qquad \text{(S3.1)}
\end{aligned}$$

Hence:

$$\begin{aligned}
\text{P}(Y_{n+k}|X_n = x_n, 1_{n+k} = 1) \sim &\text{Binomial}\left(q_{n+k}\alpha^k, x_n\right) \\
&+ \text{Poisson}\left(q_{n+k}\sum_{i=1}^{k}\alpha^{k-i}\lambda_{n+i}\right).
\end{aligned}$$

On the other hand, if $1_{n+k} = 0$, the computation are quicker, leading to:

$$\begin{aligned}
Y_{n+1} &= X_{n+1} = \alpha \circ X_n + W(\lambda_{n+1}) \\
Y_{n+2} &= X_{n+2} = \alpha \circ (\alpha \circ X_n + W(\lambda_{n+1})) + W(\lambda_{n+2}) \\
&= \alpha^2 \circ X_n + W(\alpha\lambda_{n+1}) + W(\lambda_{n+2}) \\
Y_{n+3} &= X_{n+3} = \alpha \circ (\alpha \circ (\alpha \circ X_n + W(\lambda_{n+1})) + W(\lambda_{n+2})) + W(\lambda_{n+3}) \\
&= \alpha^3 \circ X_n + W(\alpha^2\lambda_{n+1}) + W(\alpha\lambda_{n+2}) + W(\lambda_{n+3}) \\
&\vdots \\
Y_{n+k} &= \alpha^k \circ X_n + W\left(\sum_{i=1}^{k}\alpha^{k-i}\lambda_{n+i}\right) \qquad \text{(S3.2)}
\end{aligned}$$

Thus,

$$\text{P}(Y_{n+k}|X_n = x_n, 1_{n+k} = 0) \sim \text{Binomial}\left(\alpha^k, x_n\right) + \text{Poisson}\left(\sum_{i=1}^{k}\alpha^{k-i}\lambda_{n+i}\right). \qquad \text{(S3.3)}$$

Finally, the distribution of $\mathrm{P}(Y_{n+k}|X_n = x_n)$ is a mixture of two components that are a sum of a Binomial distribution and a Poisson distribution with the corresponding parameters. That is:

$$\mathrm{P}(Y_{n+k}|X_n = x_n) = \begin{cases} \text{Binomial}\left(\alpha^k, x_n\right) + \text{Poisson}\left(\sum_{i=1}^{k} \alpha^{k-i}\lambda_{n+i}\right) & 1-\omega \\ \text{Binomial}\left(q_{n+k}\alpha^k, x_n\right) + \text{Poisson}\left(q_{n+k}\sum_{i=1}^{k} \alpha^{k-i}\lambda_{n+i}\right) & \omega \end{cases}, \tag{S3.4}$$
